# Supplementary material for: Learning curve and surgical outcome of robotic assisted colorectal surgery with ERAS program
Source: Sci Rep. 2022 Nov 29;12:20566. doi: 10.1038/s41598-022-24665-w (PMC9709162; doi:10.1038/s41598-022-24665-w)
Supplement: Supplementary file 1 — Supplementary Information 1. [file 41598_2022_24665_MOESM1_ESM.zip › LSC Raw data-Ñ[▒K/060/O_ñw╝╨░O▒Kñσ.pdf]

正式摘要(電子簽章：000000183807559)

請選擇...reporter

臺中榮民總醫院

Operation Note

[redacted]

- 手術日期：2020/01/09
- 手術醫師：蔣鋒帆，CR 林昌霖，R 楊涵中，R 陳威遠
- Anesthesia : GE
- Position : Supine
- Aseptic agents : Betadine Alcohol , Betadine Aqua

Diagnosis

- Pre-operative diagnosis : A-COLON ADENOMA, FOR LAPA-OP
- Post-operative diagnosis : A-colon lateral spreading tumor, s/p laparoscopic right hemicolectomy on 20200109, cT1N0M0.

Surgical Indication

- For curative resection

Operation

- 1. Laparoscopic right hemicolectomy with side-to-side anastomosis, antiperstalsis, GIA 75/3.5*2, re-inforcement with silk (+).
  2. Vessel ligation : ileo-colic, right colic, and right branch of middle colic vessels.
  3. Cut margin: proximal (ileum) 10 cm, (colon) 6 cm, distal 13 cm.
  4. Repair of mesentery defect: nil.
  5. A 12Fr. penrose drain placed at morrison pounch.

Finding

- 1. A 3x2.5cm lateral spreading tumor, granular type at hepatic flexure. Central enduration part invade to submucosa layer. No enlarged lymph nodes at mesocolon. cT1N0M0
  2. No other gross tumor or polyp was found.
  3. There was no visible liver metastasis or visible peritoneal seeding.
  4. Omentum adhesion to A-colon.

Procedure

- General endotracheal anesthesia was induced and then the patient was placed in the supine position with legs in adducted position. Preoperative antibiotics were given. A Foley catheter and nasogastric tube were placed. The abdomen was prepped and draped in standard sterile fashion. A small vertical incision was made below the umbilicus and a 12mm trocar was inserted into the peritoneal cavity by open method. Pneumoperitoneum was established with CO2 inflation. Laparoscope was inserted and then a 12mm trocar were inserted over LUQ of abdomen. Three 5mm trocars were inserted over RLQ, RUQ and LLQ of abdomen. The liver, omentum and peritoneum were inspected for evidence of metastatic disease. No metastatic disease was noted. The small bowel was inspected and retracted to downward by grasper. The peritoneum overlying the mesentery was then scored with electrocautery and the ileocolic artery was identified, ligated with endoclips, and transected. The remaining vessels to the cecum were also divided and ligated. Using electrocautery, omentum was freed from the transverse colon and lesser sac was entered. The colon was freed from its peritoneal attachments along the line of Toldt from the cecum to the hepatic flexure. Hemostasis was checked in the operative field. Cecum, ascending colon and hepatic flexure were mobilized and then laparoscope was removed. One midline incision was made at peri-umbilical region. The ascending colon was delivered into the incision and points of transection were selected proximally and distally. Side to side anastomosis was performed with endo GIA 80/3.8. Interrupted 3-

- 0 silk Lambert sutures were used to re-inforcement at outer layer. The anastomosis was checked and found to be intact and widely patent. One 12 Fr. penrose drain was placed at anastomosis. The fascia was closed with interrupted sutures of No.1 Vicryl. The fascia of the 12 mm trochar wound was closed with 1-0 Dexon. the skin was approximated with dermabond. The patient tolerated the procedure well and was brought to the POR in stable condition.
- Estimated blood loss: minimum
- 器械數量: 正確
- 紗布數量: 正確

Image

- PAINT
  - colon & rectum

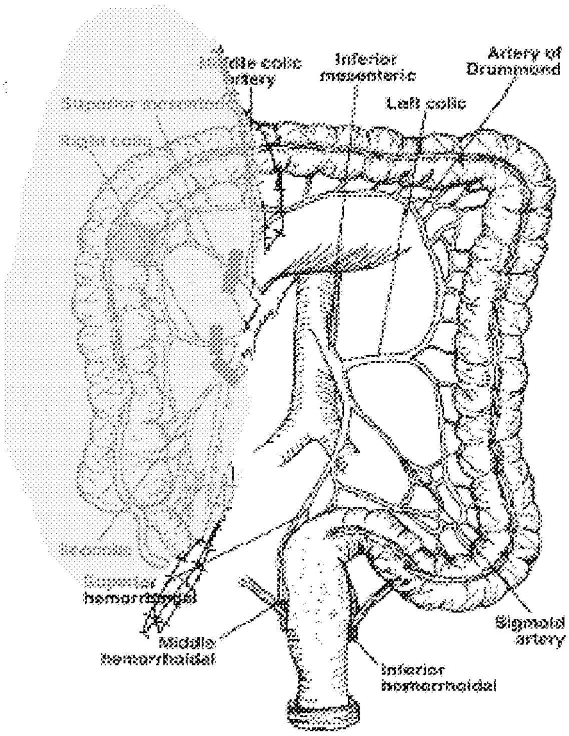

  - abdomen

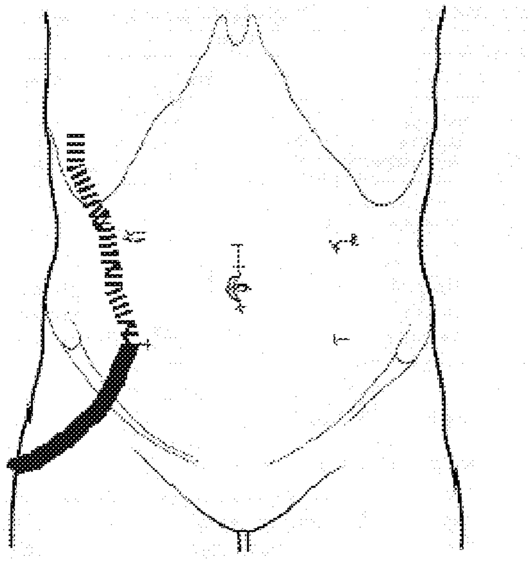

編輯主任 2062F蔣鋒帆、主治 2320B林昌霖

正式摘要(電子簽章：**000000183807559)**

請選擇...reporter

doctorRank:B
